# Association of PRECISE-HBR score with clinical outcomes and length of hospital stay after transcatheter aortic valve implantation

**DOI:** 10.55730/1300-0144.6360

**Published:** 2026-07-01

**Authors:** Erhan SARAÇOĞLU, Yunus Emre ÖZBEBEK, Bekir DEMİRTAŞ

**Affiliations:** 1 Department of Cardiology, Ankara Etlik City Hospital,Ankara, Turkiye; 2 University of Health Sciences, Ankara, Turkiye

**Keywords:** Aortic stenosis, bleeding risk, length of hospital stay, PRECISE-HBR, risk stratification, transcatheter aortic valve implantation

## Abstract

**Background/aim:**

The PRECISE-HBR score was developed to estimate bleeding risk after percutaneous coronary intervention, but its clinical utility in patients undergoing transcatheter aortic valve implantation (TAVI) remains uncertain. We evaluated its association with major bleeding and hospitalization burden after TAVI.

**Materials and methods:**

This ambispective, single-center observational study included 184 consecutive patients undergoing TAVI. The patients were categorized by PRECISE-HBR score (≤22, 23–26, and ≥27) for descriptive comparisons. Receiver operating characteristic analyses were used to assess the discriminative performance of PRECISE-HBR and the Society of Thoracic Surgeons score for major bleeding, prolonged hospitalization, and in-hospital mortality. Binary logistic regression was utilized to evaluate associations between each one-point increase in PRECISE-HBR and clinical outcomes, while correlation analyses were used to assess relationships with length of stay.

**Results:**

Higher PRECISE-HBR scores were associated with older age, female sex, hypertension, and chronic kidney disease. Major bleeding increased across score categories (7.7%, 19.4%, and 34.6%; p = 0.010) and total hospital stay was longer in the highest category (p = 0.033). PRECISE-HBR score showed weak positive correlations with intensive care unit stay (r = 0.270, p < 0.001), ward stay (r = 0.148, p = 0.044), and total hospitalization duration (r = 0.244, p = 0.001). Discrimination was moderate for major bleeding (AUC 0.748, 95% CI 0.662–0.824) and modest for prolonged hospitalization (AUC 0.615, 95% CI 0.532–0.694). Each one-point increase in PRECISE-HBR score was associated with major bleeding (OR 1.15, 95% CI 1.09–1.22; p < 0.001) and prolonged hospitalization (OR 1.06, 95% CI 1.02–1.11; p = 0.007), but not in-hospital mortality.

**Conclusion:**

Higher PRECISE-HBR scores were associated with major bleeding and longer hospitalization after TAVI. The score may aid bleeding-risk assessment and estimation of hospitalization burden, although prospective external validation is required.

## 1. Introduction

Severe aortic stenosis is a progressive and life-threatening condition associated with high morbidity and mortality if left untreated [1,2]. Transcatheter aortic valve implantation (TAVI) has emerged as an established therapeutic option across the entire surgical risk spectrum, particularly in elderly and comorbid populations [2,3]. Despite major technological and procedural advancements, TAVI patients remain at substantial risk for both thrombotic and bleeding complications, which significantly affect short- and long-term outcomes [4].

Bleeding events after TAVI are common and have been consistently associated with increased mortality, prolonged hospitalization, and impaired functional recovery. Therefore, accurate preprocedural identification of patients at high bleeding risk is essential for optimizing antithrombotic strategies and improving clinical outcomes [5]. The PRECISE-HBR score was originally proposed as a risk stratification tool to predict bleeding events in patients treated with percutaneous coronary intervention and has been validated as a reliable tool to predict bleeding risk based on routinely available clinical and laboratory parameters [6].

However, because the components of the PRECISE-HBR score include advanced age, renal dysfunction, and anemia, the score may also be associated with adverse clinical outcomes in patients undergoing TAVI [7–9]. The potential association of PRECISE-HBR score with hospitalization burden and early clinical outcomes beyond bleeding has not been adequately evaluated in patients undergoing TAVI.

In the present study, we aimed to investigate the association between PRECISE-HBR score and clinical outcomes in patients undergoing TAVI. Specifically, we evaluated its relationship with bleeding events, length of hospital stay, and early in-hospital clinical outcomes after TAVI.

## 2. Materials and methods

### 2.1. Study design and population

This ambispective, single-center observational study included consecutive patients who underwent TAVI between January 2023 and December 2025 at a tertiary referral center. A total of 184 patients were included in the final analysis.

Patients with incomplete clinical or laboratory data required for calculation of the PRECISE-HBR score were excluded. All procedures were performed according to current guideline recommendations and institutional protocols. The study was conducted in accordance with the Declaration of Helsinki and approved by the local ethics committee. Because the study database was generated from patients with complete PRECISE-HBR components, the exact number of initially screened but excluded patients could not be reliably retrieved. Therefore, the analysis was performed as a complete-case analysis of patients with available data required for PRECISE-HBR calculation.

### 2.2. Data collection and definitions

Baseline demographic characteristics, clinical comorbidities, laboratory parameters, echocardiographic findings, and procedural characteristics were obtained from hospital electronic medical records.

For each participant, the PRECISE-HBR score was calculated according to the original algorithm using seven variables: age, estimated glomerular filtration rate (eGFR), hemoglobin level, white blood cell count, previous bleeding history, long-term oral anticoagulant use, and fulfillment of the Academic Research Consortium for High Bleeding Risk (ARC-HBR) criteria. The eGFR value closest to the TAVI procedure was obtained from the institutional laboratory records and was calculated using the Chronic Kidney Disease Epidemiology Collaboration (CKD-EPI) equation. Based on the score calculated, the patients were categorized into three predefined groups (≤22, 23–26, and ≥27), in accordance with the original PRECISE-HBR derivation study.

Bleeding complications were adjudicated according to the Bleeding Academic Research Consortium (BARC) classification, and major bleeding was defined as BARC type 3 or 5 bleeding.

Chronic kidney disease was defined as an eGFR of <60 mL/min/1.73 m^2^. The predefined clinical endpoints included in-hospital stroke and in-hospital mortality. Length-of-stay parameters comprised intensive care unit stay, ward stay, and total hospitalization duration. Access-route data and vascular complications were not uniformly available in the study database and therefore could not be incorporated into the adjusted analyses.

### 2.3. Procedural details

All TAVI procedures were performed by experienced heart teams using standard techniques and commercially available transcatheter valve systems. Valve type and size were selected based on preprocedural imaging and anatomical suitability. Procedural and postprocedural management followed current guideline-based practices.

### 2.4. Statistical analysis

Statistical analyses were performed using SPSS version 29.0 (IBM Corp., Armonk, NY, USA). Data distribution was evaluated with the Kolmogorov–Smirnov test. Continuous variables were expressed as mean ± standard deviation or median (range), whereas categorical variables were presented as counts and percentages.

Group comparisons were performed using one-way ANOVA or the Kruskal–Wallis test for continuous variables and the chi-squared or Fisher’s exact test for categorical variables. Spearman’s correlation analysis was used to evaluate the association between PRECISE-HBR score and hospital length of stay. Receiver operating characteristic curve analyses were performed to evaluate the discriminative performance of the PRECISE-HBR and Society of Thoracic Surgeons (STS) scores for major bleeding, prolonged hospitalization, and in-hospital mortality. Areas under the curve with 95% confidence intervals were calculated. Binary logistic regression analyses were used to estimate the odds ratios and 95% confidence intervals associated with each one-point increase in the PRECISE-HBR score. Prolonged hospitalization was defined as a total hospital stay of ≥14 days, corresponding to a stay above the cohort median. The incremental value of PRECISE-HBR beyond the STS score was assessed using likelihood-ratio testing after adding PRECISE-HBR to a baseline model containing the STS score. Because the analyses were exploratory and hypothesis-generating, no formal adjustment for multiple comparisons was applied; therefore, p-values should be interpreted with caution.

### 2.5. Ethical approval

The study was approved by the Ankara Etlik City Hospital No. 2 Scientific Research Evaluation and Ethics Committee (approval reference number: AEŞH-BADEK2-2025-333; approval date: 19 August 2025) and was conducted in accordance with the Declaration of Helsinki. The study was initially approved as a prospective descriptive observational study. During data collection, it was determined that all study variables were routinely available in the institutional electronic medical records without requiring additional patient contact, intervention, or diagnostic testing. Therefore, eligible consecutive TAVI patients from the same study framework and data structure were identified from existing institutional records and included in the final analysis.

## 3. Results

A total of 184 patients who underwent transcatheter aortic valve implantation were included in the final analysis. The baseline demographic, clinical, laboratory, and procedural characteristics are summarized in Table 1. The mean age was 78.9 ± 8.1 years and 107 patients (58.2%) were female. Hypertension was the most common comorbidity (77.7%), followed by diabetes mellitus (37.0%), chronic obstructive pulmonary disease (22.8%), prior percutaneous coronary intervention (22.3%), prior coronary artery bypass grafting (16.3%), and chronic kidney disease (14.8%). The mean STS score was 5.83 ± 2.85% and the mean left ventricular ejection fraction was 52.2 ± 11.67%. The mean PRECISE-HBR score was 30.26 ± 7.28 and 127 patients (69.0%) were classified in the highest PRECISE-HBR category (≥27). Major bleeding, defined as BARC type 3 or 5 bleeding, occurred in 52 patients (28.3%). In-hospital stroke and in-hospital mortality occurred in 11 patients (6.0%) and 10 patients (5.4%), respectively.

Comparisons according to PRECISE-HBR categories are presented in Table 2. The patients in higher PRECISE-HBR categories were significantly older, with median ages of 72.5, 78, and 81 years in the ≤22, 23–26, and ≥27 groups, respectively (p < 0.001). The proportion of female patients increased progressively across the categories (26.9%, 41.9%, and 68.5%, respectively; p < 0.001). Hypertension and chronic kidney disease were more frequent in the patients with higher PRECISE-HBR scores (p = 0.011 and p = 0.019, respectively). STS score increased significantly across the PRECISE-HBR categories (p = 0.004), whereas left ventricular ejection fraction did not differ significantly between the groups (p = 0.955).

Laboratory parameters also differed across the PRECISE-HBR categories. Hemoglobin levels decreased progressively from the ≤22 group to the ≥27 group (14.13 ± 0.93, 12.81 ± 1.53, and 10.70 ± 1.88 g/dL, respectively; p < 0.001). Glomerular filtration rate was significantly lower in the highest PRECISE-HBR category (p < 0.001). Creatinine, albumin, and red cell distribution width values also differed significantly across the categories (p = 0.008, p = 0.014, and p < 0.001, respectively).

Major bleeding increased significantly across the PRECISE-HBR categories, occurring in two patients (7.7%) in the ≤22 group, six patients (19.4%) in the 23–26 group, and 44 patients (34.6%) in the ≥27 group (p = 0.010). In-hospital stroke and in-hospital mortality did not differ significantly between the PRECISE-HBR categories (p = 0.232 and p = 0.280, respectively). Total hospital stay was significantly longer in the patients with higher PRECISE-HBR scores, with median values of 11.5, 11, and 14 days in the ≤22, 23–26, and ≥27 groups, respectively (p = 0.033).

The discriminative performance of the PRECISE-HBR and STS scores is shown in Table 3. The ROC analysis demonstrated that the PRECISE-HBR score had moderate discriminative ability for major bleeding (AUC 0.748, 95% CI 0.662–0.824), modest discriminative ability for prolonged hospitalization (AUC 0.615, 95% CI 0.532–0.694), and limited discriminative ability for in-hospital mortality (AUC 0.547, 95% CI 0.372–0.713). The corresponding AUC values for the STS score were 0.634 (95% CI 0.538–0.726), 0.581 (95% CI 0.501–0.664), and 0.447 (95% CI 0.311–0.609), respectively. When analyzed as a continuous variable, each one-point increase in PRECISE-HBR score was associated with higher odds of major bleeding (OR 1.15, 95% CI 1.09–1.22, p < 0.001) and prolonged hospitalization (OR 1.06, 95% CI 1.02–1.11, p = 0.007), but not with in-hospital mortality (OR 1.02, 95% CI 0.93–1.11, p = 0.672). Adding PRECISE-HBR to a baseline model containing the STS score significantly improved model fit for major bleeding (LR χ^2^ = 21.67, p < 0.001) and prolonged hospitalization (LR χ^2^ = 4.48, p = 0.034), but not for in-hospital mortality (LR χ^2^ = 0.13, p = 0.715). The ROC curve demonstrating the discriminative performance of the PRECISE-HBR score for major bleeding is presented in the Figure.

The relationship between PRECISE-HBR score and length of stay is presented in Table 4. PRECISE-HBR score showed weak but statistically significant positive correlations with intensive care unit stay (Spearman’s r = 0.270, p < 0.001), ward stay (r = 0.148, p = 0.044), and total hospital stay (r = 0.244, p = 0.001). These findings indicate weak but statistically significant positive associations between higher PRECISE-HBR scores and longer hospitalization after TAVI.

## 4. Discussion

In the present study, we found that higher PRECISE-HBR scores were associated with increased major bleeding and longer hospitalization in patients undergoing TAVI. The principal findings of our study are as follows: (i) higher PRECISE-HBR scores were associated with older age, female sex, higher prevalence of hypertension and chronic kidney disease, and lower hemoglobin levels; (ii) major bleeding events increased significantly with increasing PRECISE-HBR categories; (iii) higher PRECISE-HBR scores were associated with longer ICU and total hospital stay.

Importantly, hard clinical endpoints, including in-hospital stroke and in-hospital mortality, did not differ significantly across the PRECISE-HBR categories. Therefore, our findings should not be interpreted as evidence that PRECISE-HBR score predicts mortality after TAVI. The limited number of fatal events may have reduced the statistical power to detect such associations. Accordingly, PRECISE-HBR score should be considered mainly a bleeding-risk and hospitalization-burden marker rather than a comprehensive prognostic tool for mortality.

Bleeding complications remain a major concern after TAVI and have been consistently associated with increased mortality and worse clinical outcomes [10,11]. Previous studies have shown that bleeding risk scores developed for percutaneous coronary interventions, including PRECISE-HBR, may also be useful in structural heart interventions [7,9,12]. Renal impairment and baseline anemia are also recognized as clinically relevant risk markers in patients undergoing TAVI [13,14]. Dedicated high-bleeding-risk criteria have also been evaluated in TAVI populations, further supporting the importance of structured bleeding-risk assessment in this setting [15]. Our findings support this concept by demonstrating a clear stepwise increase in major bleeding rates across PRECISE-HBR categories in the TAVI population. Importantly, the magnitude of bleeding risk observed in our cohort is consistent with prior real-world data, reinforcing the validity of PRECISE-HBR in this setting. The relatively high rate of major bleeding observed in our cohort should be interpreted in the context of an elderly and highly comorbid TAVI population.

One of the most clinically relevant findings of our study is the association between PRECISE-HBR score and length of hospital stay. Other procedure-related factors, including conduction disturbances and the need for permanent pacemaker implantation, may also influence post-TAVI monitoring and hospitalization [16]. We observed a significant positive correlation between PRECISE-HBR score and ICU stay, ward stay, and total hospitalization duration. This suggests that PRECISE-HBR score may serve not only as a bleeding risk tool but also as a practical indicator of resource utilization and postprocedural recovery. From a clinical perspective, early identification of high PRECISE-HBR patients may help clinicians anticipate more complex periprocedural management, optimize postprocedural monitoring, and improve discharge planning. Although the observed correlations were statistically significant, their magnitude was modest, suggesting that PRECISE-HBR score represents only one of several determinants of postprocedural length of stay.

Because categorization of continuous risk scores may lead to information loss and reduced statistical power, we additionally analyzed PRECISE-HBR score as a continuous variable. The associations observed between increasing PRECISE-HBR values and both major bleeding and prolonged hospitalization support the robustness of our findings beyond the predefined score categories.

The clinical implications of our findings are important. PRECISE-HBR is a simple and readily available score that can be calculated using routine clinical and laboratory data [6]. Incorporating PRECISE-HBR score into the preprocedural assessment of TAVI patients may provide additional information for bleeding risk assessment and postprocedural management and may help clinicians identify patients who require closer bleeding surveillance, careful review of antithrombotic therapy, and individualized discharge planning. The high proportion of patients in the ≥27 category likely reflects the advanced age and comorbidity burden of contemporary TAVI populations.

Several limitations of the present study should be acknowledged. First, this was an ambispective, single-center observational study with a relatively limited sample size, which may restrict the generalizability of the findings. Second, because the study database was generated from patients with complete PRECISE-HBR components, the exact number of initially screened but excluded patients could not be reliably retrieved; therefore, a complete-case analysis was performed. Third, residual confounding due to unmeasured variables cannot be excluded. In particular, access route and vascular complications, which are important determinants of bleeding after TAVI, were not systematically available in the dataset and could not be incorporated into the analyses. Similarly, although postprocedural oral anticoagulant and antithrombotic regimens may influence bleeding risk, the present study was not designed to evaluate their independent effects on bleeding outcomes. Fourth, frailty was not formally assessed using validated frailty instruments; therefore, no direct conclusions can be drawn regarding the relationship between PRECISE-HBR score and frailty status. Fifth, because the lower PRECISE-HBR categories included relatively small numbers of patients, category-based multivariable modeling was avoided to reduce the risk of overfitting and unstable estimates. Although predefined PRECISE-HBR categories were used to facilitate descriptive comparisons, categorization of a continuous score may result in information loss. Sixth, no formal a priori sample-size calculation was performed, as the study included all eligible patients available during the study period, and the limited number of mortality events may have reduced the statistical power to detect differences in hard clinical endpoints. Seventh, formal calibration and net reclassification improvement analyses were not performed because outcome-specific predicted probabilities and validated risk categories for PRECISE-HBR have not been established in the TAVI population, and the limited number of events could have resulted in unstable estimates. Eighth, because no formal adjustment for multiple comparisons was applied, the possibility of type I error inflation should be considered. Finally, long-term outcomes were not available; therefore, the long-term prognostic value of PRECISE-HBR score could not be assessed. Larger, multicenter prospective studies are needed to validate the clinical utility of PRECISE-HBR score in patients undergoing TAVI.

## 5. Conclusion

Higher PRECISE-HBR scores were associated with an increased risk of major bleeding and longer hospitalization in patients undergoing TAVI. However, in-hospital stroke and in-hospital mortality did not differ significantly across the PRECISE-HBR categories. These findings support the potential utility of PRECISE-HBR score for bleeding risk assessment and estimation of hospitalization burden, but not as a validated mortality prediction tool in TAVI patients.

## Figures and Tables

**Figure:**
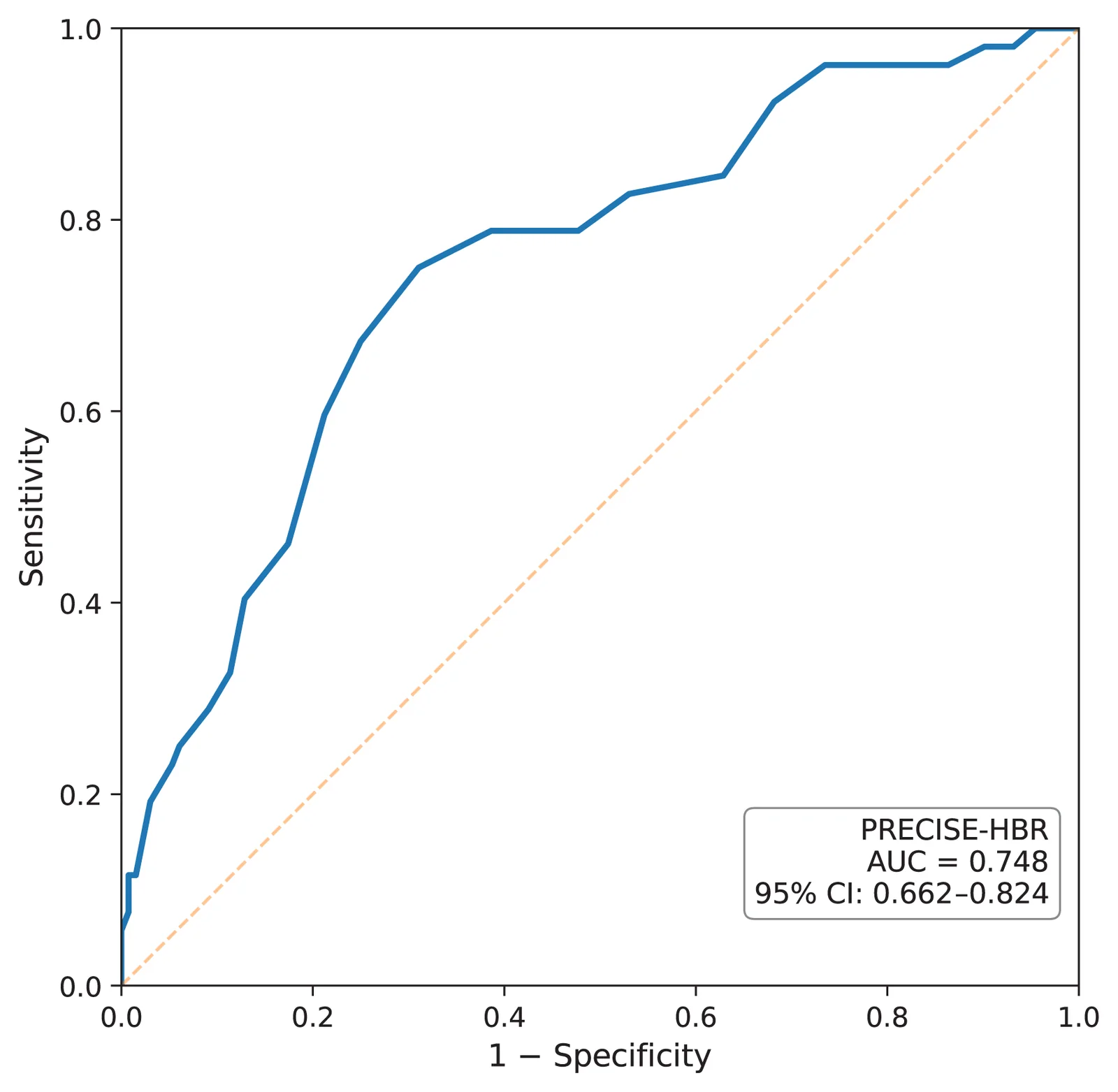
Receiver operating characteristic curve demonstrating the discriminative performance of the PRECISE-HBR score for major bleeding, defined as Bleeding Academic Research Consortium type 3 or 5 bleeding, after transcatheter aortic valve implantation. The area under the curve was 0.748 (95% confidence interval, 0.662–0.824). AUC: area under the curve; BARC: Bleeding Academic Research Consortium; CI: confidence interval; PRECISE-HBR: Predicting Bleeding Complications in Patients Undergoing Stent Implantation and Subsequent Dual Antiplatelet Therapy–High Bleeding Risk.

**Table 1:** Baseline demographic, clinical, laboratory, and procedural characteristics of the study population undergoing TAVI.

Variable	n (%) or mean ± SD	Median (min–max)
**Demographic and clinical characteristics**		
Age (years)	78.9 ± 8.1	79.5 (63–97)
Female sex	107 (58.2)	–
Diabetes mellitus	68 (37.0)	–
Hypertension	143 (77.7)	–
Chronic kidney disease	27 (14.8)	–
Chronic obstructive pulmonary disease	42 (22.8)	–
Prior PCI	41 (22.3)	–
Prior CABG	30 (16.3)	–
History of bleeding	7 (3.8)	–
STS score (%)	5.83 ± 2.85	5 (1–17)
LVEF (%)	52.2 ± 11.67	56 (15–65)
**Aortic stenosis type**		
High-gradient AS	161/182 (88.5)	–
Low-flow, low-gradient AS	21/182 (11.5)	–
**Valve morphology**		
Tricuspid valve	178/183 (97.3)	–
Bicuspid valve	2/183 (1.1)	–
Bioprosthetic valve	3/183 (1.6)	–
**Antithrombotic therapy** *		
Clopidogrel	130 (70.7)	–
Oral anticoagulant (any)	39 (21.2)	–
Acetylsalicylic acid	16 (8.7)	–
**PRECISE-HBR score**		
PRECISE-HBR score	30.26 ± 7.28	30.5 (9–52)
≤22	26 (14.1)	–
23–26	31 (16.8)	–
≥27	127 (69.0)	–
**Key laboratory parameters**		
Hemoglobin (g/dL)	11.54 ± 2.16	11.7 (5.2–15.9)
Creatinine (mg/dL)	1.14 ± 0.65	1.03 (0.41–7.25)
GFR (mL/min/1.73 m^2^)	59.54 ± 21.89	58 (5–130)
Albumin (g/dL)	3.84 ± 0.44	3.85 (2.4–5.35)
**Procedural outcomes**		
Permanent pacemaker implantation	28 (15.3)	–
Major bleeding	52 (28.3)	–
In-hospital stroke	11 (6.0)	–
In-hospital mortality	10 (5.4)	–
ICU stay (days)	4.39 ± 5.11	3 (0–40)
Total hospital stay (days)	15.02 ± 8.78	13.5 (1–62)

Data are presented as mean ± standard deviation (SD), median (minimum–maximum), or number (percentage), as appropriate. Denominators varied due to missing data; percentages were calculated using available data for each variable.

* Antithrombotic therapy refers to postprocedural discharge medications. Combination antithrombotic therapy was permitted; therefore, percentages may exceed 100%.

AS: aortic stenosis; CABG: coronary artery bypass grafting; CKD: chronic kidney disease; COPD: chronic obstructive pulmonary disease; GFR: glomerular filtration rate; HBR: high bleeding risk; ICU: intensive care unit; LVEF: left ventricular ejection fraction; PCI: percutaneous coronary intervention; STS: Society of Thoracic Surgeons; TAVI: transcatheter aortic valve implantation.

**Table 2:** Comparison of demographic, clinical, laboratory, and outcome characteristics according to PRECISE-HBR categories.

Variable	≤22 (n = 26)	23–26 (n = 31)	≥27 (n = 127)	p value
**Demographic and clinical characteristics**				
Age, years	72.5 (63–91)	78 (64–93)	81 (64–97)	<0.001
Female sex, n (%)	7 (26.9)	13 (41.9)	87 (68.5)	<0.001
Diabetes mellitus, n (%)	7 (26.9)	11 (35.5)	50 (39.4)	0.480
Hypertension, n (%)	15 (57.7)	22 (71.0)	106 (83.5)	0.011
Chronic kidney disease, n (%)	0 (0)	3 (9.7)	24 (19.0)	0.019
Prior CABG, n (%)	8 (30.8)	7 (22.6)	15 (11.8)	0.035
STS score (%)	4 (1–8)	5 (1–14)	6 (1–17)	0.004
LVEF (%)	60 (15–65)	60 (15–65)	55 (20–65)	0.955
**Key laboratory parameters**				
Hemoglobin (g/dL)	14.13 ± 0.93	12.81 ± 1.53	10.70 ± 1.88	<0.001
Creatinine (mg/dL)	0.97 (0.55–1.46)	0.92 (0.41–1.53)	1.09 (0.43–7.25)	0.008
GFR (mL/min/1.73 m²)	71.65 ± 19.17	71.39 ± 19.51	54.17 ± 20.94	<0.001
Albumin (g/dL)	4.10 (2.93–4.97)	3.92 (2.97–4.56)	3.80 (2.40–5.35)	0.014
RDW (%)	13.65 (12.7–20.8)	14.2 (11.9–31.7)	15.1 (12.4–46)	<0.001
**Procedural and clinical outcomes**				
Major bleeding, n (%)	2 (7.7)	6 (19.4)	44 (34.6)	0.010
In-hospital stroke, n (%)	1 (3.8)	0 (0)	10 (7.9)	0.232
In-hospital mortality, n (%)	0 (0)	3 (9.7)	7 (5.5)	0.280
Total hospital stay, days	11.5 (4–49)	11 (1–41)	14 (3–62)	0.033

Data are presented as mean ± standard deviation (SD), median (minimum–maximum), or number (percentage), as appropriate; p-values were obtained using one-way ANOVA or the Kruskal–Wallis test for continuous variables and the chi-squared or Fisher exact test for categorical variables. CABG: coronary artery bypass grafting; GFR: glomerular filtration rate; HBR: high bleeding risk; LVEF: left ventricular ejection fraction; RDW: red cell distribution width; STS: Society of Thoracic Surgeons.

**Table 3:** Discriminative performance and logistic regression analyses of PRECISE-HBR score for clinical outcomes.

Outcome	PRECISE-HBR AUC (95% CI)	STS AUC (95% CI)	OR per 1-point PRECISE-HBR increase (95% CI)	p value	Incremental value beyond STS, LR χ²	p value
**Major bleeding**	0.748 (0.662–0.824)	0.634 (0.538–0.726)	1.15 (1.09–1.22)	<0.001	21.67	<0.001
**Prolonged hospitalization**	0.615 (0.532–0.694)	0.581 (0.501–0.664)	1.06 (1.02–1.11)	0.007	4.48	0.034
**In-hospital mortality**	0.547 (0.372–0.713)	0.447 (0.311–0.609)	1.02 (0.93–1.11)	0.672	0.13	0.715

AUC: area under the receiver operating characteristic curve; CI: confidence interval; HBR: high bleeding risk; LR: likelihood-ratio; OR: odds ratio; PRECISE-HBR: Predicting Bleeding Complications in Patients Undergoing Stent Implantation and Subsequent Dual Antiplatelet Therapy–High Bleeding Risk; STS: Society of Thoracic Surgeons. Prolonged hospitalization was defined as total hospital stay ≥14 days, corresponding to a stay above the cohort median. Incremental value beyond STS was assessed by likelihood-ratio testing after adding PRECISE-HBR to a baseline model including STS score.

**Table 4:** Correlation between PRECISE-HBR score and length of stay.

Variable	Spearman’s r	p value
ICU stay (days)	0.270	<0.001
Ward stay (days)	0.148	0.044
Total hospital stay (days)	0.244	0.001

Spearman’s rank correlation coefficient was used. HBR: high bleeding risk; ICU: intensive care unit.

## Data Availability

The data supporting the findings of this study are available from the corresponding author upon reasonable request.
